# The connection between diverticulosis and colonic superficial neoplastic lesions in patients who underwent screening colonoscopy

**DOI:** 10.1007/s00384-023-04399-5

**Published:** 2023-04-21

**Authors:** M. Valvano, V. Vezzaro, S. Fabiani, A. Capannolo, E. Sgamma, N. Cesaro, G. Valerii, S. Longo, C. Barbera, L. Lombardi, A. Viscido, S. Necozione, G. Latella

**Affiliations:** 1 Division of Gastroenterology, Hepatology, and Nutrition, Department of Life, Health and Environmental Sciences, University of L’Aquila, Piazzale Salvatore Tommasi 1, 67100 L’Aquila, Italy; 2 Diagnostic and Surgical Endoscopy Unit, San Salvatore Academic Hospital, L’Aquila, Italy; 3 Gastroenterology and Endoscopy Unit, Ospedale G. Mazzini, 64100 Teramo, Italy; 4 Epidemiology Unit, Department of Life, Health and Environmental Sciences, University of L’Aquila, L’Aquila, Italy

**Keywords:** Diverticulosis, Colonic diverticula, Colonic polyps, Colorectal cancer

## Abstract

**Purpose:**

If could be a potential pathophysiological connection between colonic diverticula and colonic superficial neoplastic lesions, beyond the shared risk factors, has been a subject of debate in the last years. This study tries to evaluate the association between diverticulosis and colonic neoplastic lesions.

**Methods:**

This is a cross-sectional study including asymptomatic patients who underwent a screening colonoscopy (patients with a positive fecal occult blood test under the regional program of colorectal cancer (CRC) screening), surveillance after polypectomy resection, or familiarity (first-degree relatives) between 2020 and 2021 to evaluate the association between diverticula and colonic polyps. A multivariate analysis with multiple logistic regression and odds ratio (OR) to study the independent association between adenomas and adenocarcinomas was performed.

**Results:**

One thousand five hundred one patients were included. A statistically significant association between adenomas or CRC alone and colonic diverticula was found (*p* = 0.045). On a multivariate analysis of demographic (age, gender) and clinical parameters (familiarity for diverticula and adenoma/CRC), only age was significantly associated with the development of colorectal adenomas or cancer (OR 1.05, 95% CI 1.03–1.07, *p* < 0.0001).

**Conclusions:**

This study showed a statistically significant association between diverticula and colonic adenomas. However, it is impossible to establish a cause-effect relationship due to the intrinsic characteristics of this study design. A study with a prospective design including both patients with diverticulosis and without colonic diverticula aimed at establishing the incidence of adenoma and CRC could help to answer this relevant clinical question, since a potential association could indicate the need for closer endoscopic surveillance.

## Introduction

Colonic diverticula and colonic superficial neoplastic lesions (both polypoid and non-polypoid) are the most common findings during a screening colonoscopy [1, 2]. If could be a potential pathophysiological connection between these two findings, beyond the shared risk factors, has been a subject of debate in the last few years [3].

Colonic diverticula are blind-ended pouches of the mucosa and submucosa through the tunica muscle [4]. The term diverticulosis refers to the presence of one or more diverticula in absence of gastrointestinal symptoms or inflammation of the peri-diverticular mucosa. Thus, the diverticular disease can be categorized as symptomatic or asymptomatic, complicated, or uncomplicated [5]. The presence of associated symptoms such as abdominal pain, bloating, diarrhea, and constipation in absence of inflammation of peri-diverticular mucosa identifies symptomatic uncomplicated diverticular disease (SUDD) [4, 6].

On the other hand, segmental colitis associated with diverticulosis (SCAD) is characterized by inflammation of peri-diverticular mucosa and acute abdominal pain and bloody diarrhea, often combined with an increase in erythrocyte sedimentation rate (ESR), C-reactive protein (CRP), leukocytes, and  faecal calprotectin [7].

Moreover, diverticulitis is described as a localized inflammatory response that develops following a micro-perforation of the diverticular fundus or following the entrapment of a coprolite in a diverticulum and subsequent exposure of the lamina propria to the microbiota [8]. This condition is the most usual clinical complication of diverticulosis, and the clinical presentation is characterized by acute pain often accompanied by fever, alterations of the bowel habit, and urinary symptoms such as dysuria, pollakiuria, and bladder tenesmus [9]. In addition, diverticulitis could be followed by complications, such as perforations, abscesses, fistulas, and strictures in about 20% of cases [10].

Additionally, diverticular bleeding (DB) is the most common cause of lower gastrointestinal (GI) bleeding and the most frequent complication of diverticular disease [11]. It is mostly self-limited, and painless and the clinical presentation is characterized by massive rectal bleeding. It is not necessarily associated with local inflammation or episodes of diverticulitis, but it is caused by the rupture of diverticula-associated arteries [12].

Some commonly used drugs, especially in elderly patients, can be trigger factors of DB episodes such as anticoagulants, antiplatelet agents, and NSAIDs [13, 14].

Several studies showed that the prevalence of diverticulosis increases with age and has a geographical distribution; in fact, it is more prevalent in Western countries, compared to Africa and Asia [15, 16]. The geographical distribution of diverticulosis and the increase of the same with age could be due to the Western diet, lacking fibers, which leads to an increased time of intestinal transit and a consequent increase in colonic intraluminal pressure during defecation [17–20].

As recommended by the European Society of Gastrointestinal Endoscopy (ESGE), the morphology of the colonic superficial neoplastic lesions should be described using the Paris classification system [21, 22].

The superficial neoplastic lesions are classified endoscopically into polypoid and non-polypoid lesions, and pathologically into adenomatous, hamartomatous, inflammatory, and hyperplastic polyps [23].

Colorectal cancer (CRC) represents 10% of all cancers, and it is the second and third cause of cancer in women and men respectively [24, 25].

Death due to CRC is decreasing, with rates diminished by about 10% in the last 5 years [26].

These advances are mainly imputable to colonoscopy screening programs, early diagnosis, and improvement of medical and surgical therapies. Also important is the continuous improvement of endoscopic techniques for the detection and resection of polyps, despite some complications such as perforation and bleeding, the latter, especially in some patients on anticoagulant and antiplatelet therapy [27].

Despite this, CRC still represents one of the major causes of death in the world.

Among the modifiable environmental risk factors, diet plays a primary role in etiology. A diet high in red meat and animal fat is correlated with an increased risk of CRC [28].

Despite the widespread use of screening colonoscopy and the wide availability of data, there is conflicting evidence regarding the association between diverticulosis and the development of polyps and/or CRC [29, 30]. Given that the prevalence of both has increased in recent years and given the common risk factors, various studies have been conducted to highlight a possible association between the two conditions, obtaining conflicting results [31, 32].

Some studies have found an association between colonic diverticula and a higher incidence of neoplastic lesions of the colon, while others have excluded it [30, 33].

This cross-sectional study aims to establish the relationship between diverticulosis and adenoma/CRC in patients undergoing screening colonoscopy since this could have important implications for CRC screening programs.

## Materials and methods

This is a cross-sectional study including all the asymptomatic patients who underwent a screening colonoscopy between 2020 and 2021.

All clinical investigations were conducted according to the principles laid down in the Declaration of Helsinki and reported according to the Strengthening the Reporting of Observational Studies in Epidemiology (STROBE) Statement guidelines [34]. Ethics approval was issued by the Internal Review Board of the University of L’Aquila (protocol number 37/2018). Informed consent was obtained from all subjects for participation in the current study.

### Inclusion and exclusion criteria

#### Inclusion criteria

- First-time colonoscopy screening
- Positive fecal occult blood test under the regional program of CRC screening
- Surveillance after polypectomy resection in a previous colonoscopy in the previous 5 years
- Familiarity (first-degree relatives)

### Exclusion criteria

- Previous colonic resection
- Inflammatory bowel disease
- Inadequate bowel preparation (Boston scale < 6 or < 2 in any of the colonic tract) [35]
- Ischemic or infective colitis
- Incomplete examination (absence of caecal intubation) [36]

All the colonoscopies performed with the above-mentioned criteria at the Gastroenterology, Hepatology, and Nutrition Division of the University of L’Aquila; Diagnostic and Surgical Endoscopy Unit, San Salvatore Academic Hospital, L'Aquila; and Gastroenterology and Endoscopy Unit, G. Mazzini Hospital, Teramo, were included.

### Study procedure

All the patients took high-volume or low-volume PEG-based regimens before the colonoscopy, following the ESGE guidelines [35].

All the exams were performed under sedation with midazolam alone or combined with fentanyl.

All the medical records were recorded with a standardized report system according to the current guidelines [36].

All the identified polypoid lesions were removed and retrieved (if it was possible) for histological analysis [21, 36]. In the case of non-resectable neoplastic lesions, biopsy sampling was performed.

Histological samples were analyzed by an expert pathologist according to the hospital protocol.

### Medical records

- Collected data of polypoid or non-polypoid lesions: number, localization, morphology, size (in millimeters), and surface pattern [21, 22].
- Collected data of diverticula: number, localization, size, and complication [37].

Diverticulosis was defined as the asymptomatic presence of diverticula (in absence of any typical gastrointestinal symptom, such as bloating, abdominal pain, irregular bowel habits, or rectal bleeding) linked to the indication of the colonoscopy examination.

### Histological classification

The retrieved lesions were categorized as adenoma, carcinoma, hyperplastic or inflammatory polyps.

### Statistical analysis

Data were compared using the *chi-square* or *Fisher’s exact test* as appropriate for the dichotomous variables. Continuous variables were reported as means with standard deviations (± SD) or as median and range. To evaluate the association between the prevalence of polyps or CRC and diverticulosis, the relative risk (RR) with a 95% CI was evaluated.

A multivariate analysis with multiple logistic regression and odds ratio (OR) adjusted for age, gender, family history for CRC, family history for diverticula, and presence of diverticula to study the independent association between adenomas and adenocarcinomas was performed. Only adenoma and carcinoma were evaluated in the multivariate analysis to assess the relationship between diverticula and colorectal superficial lesions with malignant potential.

Results were considered statistically significant at the *p* < 0.05 level.

All the statistical analyses were performed with the statistical software STATA 15.1 2017 (StataCorp LLC, College Station, TX, USA).

## Results

### Included population

A total of 1501 patients were included: 473 (31.45%) provided by the Unit of Gastroenterology, Hepatology, and Nutrition of San Salvatore Hospital, 487 (32.45%) by the Unit of Endoscopic Surgery of San Salvatore Hospital based in L’Aquila, and 541 (36.04%) by Unit of Gastroenterology and Digestive Endoscopy of Giuseppe Mazzini Hospital based in Teramo.

The study population was divided into four groups (Table 1): Group A, including 259 patients (17.26%) had only diverticulosis; Group B, 459 patients (30.58%) had only polyps or CRC; Group C, 268 patients (17.85%) had polyps or CRC and diverticulosis; Group D, 515 patients (34.31%) without superficial colonic lesions or diverticulosis.

**Table 1:** Clinical characteristics of patients included in the study

	**Group A** **Diverticulosis** *N*. 259	**Group B** **Polyps/CRC** *N*. 459	**Group C** **Polyps/CRC and diverticulosis** *N*. 268	**Group D** **Absence of diverticula and polyps/CRC** *N*. 515
**Mean age** **(range)**	68 (34–90)	63 (26–86)	68 (40–93)	60 (29–89)
**Sex** ** Female** ***N* (%)** ** Male** ***N*** **(%)**	142 (54.83%) 117 (45.17%)	206 (44.88%) 253 (55.12%)	104 (38.81%) 164 (61.19%)	308 (59.81%) 207 (40.19%)
Familiarity for CRC* ** Negative** ***N*** **(%)** ** Positive** ***N*** **(%)**	24 (53.33%) 21 (46.67%)	91 (63.64%) 52 (36.36%)	72 (75.79%) 23 (24.21%)	43 (47.25%) 48 (52.75%)
Familiarity for diverticulosis** ** Negative** ***N*** **(%)** ** Positive** ***N*** **(%)**	36 (100%) 0	119 (96.75%) 4 (3.25%)	86 (96.63%) 3 (3.37%)	72 (100%) 0

*CRC* colorectal cancer

*frequency missing = 1127

**frequency missing = 1181

The baseline characteristics of people with a potentially malignant lesion (adenoma) or carcinoma are reported in Table 2.

**Table 2:** Baseline characteristics of people with adenoma or carcinoma

	**DIVERTICULOSIS** **(*N*: 203)**			**ABSENCE OF DIVERTICULA** **(*N*: 322)**		
Hystological type	**Adenoma** **191 (94%)**	**CRC** **8 (4%)**	**Adenoma and CRC** **4 (2%)**	**Adenoma** **299 (93%)**	**CRC** **19 (6%)**	**Adenoma and CRC** **4 (1%)**
**Mean age (range)**	68 (40–88)	74 (52–89)	82 (69–93)	63 (26–86)	67 (43–85)	66 (59–70)
**Sex** ** Female** ***N*** **(%)** ** Male** ***N* (%)**	76 (40%) 115 (60%)	5 (62%) 3 (38%)	2 (50%) 2 (50%)	136 (45%) 163 (55%)	11 (58%) 8 (42%)	0 (0%) 4 (100%)

*CRC* colorectal cancer

The mean age of the study population was 63.3 years (SD ± 10.4); the minimum age was 26 years while the maximum age was 93 years. Of these 1501 patients, 760 (50.63%) were female and 741 (49.37%) were male.

### Prevalence of diverticulosis and superficial colonic lesions

**Fig. 1:**
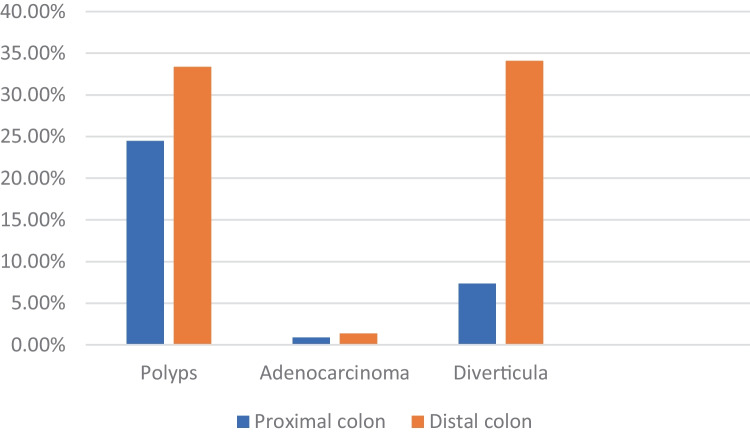
Distribution of diverticula and neoplastic lesions in proximal and distal colon

Of this, 65.76% (986/1501) had at least one lesion among diverticula or polyps/CRC, in particular: 35.11% (527/1501) presented diverticulosis, 2.35% (34/1501) CRC, and 47.04% (706/1501) polyps. Diverticulosis, polyps, and CRC were found more frequently in the distal colon, compared to the proximal colon (Fig. 1).

Regarding the histological type of polyps, 35.11% (507/1501) of patients had adenomas, 15.93% (230/1501) hyperplastic polyps, and 0.97% (14/1501) inflammatory polyps. For Group C, the results are shown in Table 3.

**Table 3:** Histological features and localization of colonic superficial lesions associated with diverticula (Group C patients)

**Histology of polyps/CRC**				
**Localization of diverticula in the colon tracts**	Inflammatory polyps* N (%) 7 (2.36%)	Hyperplastic polyps* N (%) 80 (27.03%)	Adenomas* N (%) 198 (66.89%)	CRC* N (%) 11 (3.72%)
**Proximal colon**	0	1 (1.25%)	7 (3.54%)	0
**Distal colon**	6 (85.71%)	55 (68.75%)	156 (78.79%)	10 (90.91%)
**Proximal and distal colon**	1 (14.29%)	24 (30%)	35 (17.68%)	1 (9.09%)

*CRC* colorectal cancer

*frequency missing = 17

In 57 patients, retrieving the removed lesions for histological analysis was not possible.

### Correlation between diverticulosis and colonic superficial lesions

There was no statistically significant increase in RR in the association between all types of polyps or CRC and diverticula (*p* = 0.18; Table 4).

**Table 4:** The relative risk of the association between colonic superficial lesions or cancer with colonic diverticula

**Polyps/CRC**	**Diverticula**		**Total**
	**Presence**	**Absence**	
**Absence**	259	514	773 (51.50%)
**Presence**	268	460	728 (48.50%)
**Total**	527	974	1501
**Relative risk (RR)** 1.07			
**CI** (0.97–1.19)			
***p* value** 0.18			

*CRC* colorectal cancer

On the other hand, a statistically significant association between adenomas or CRC alone and colonic diverticula were found (*p* = 0.045; Table 5).

**Table 5:** The relative risk of the association between adenoma and cancer with colonic diverticula

**Adenoma/CRC**	**Diverticula**		**Total**
	**Presence**	**Absence**	
**Absence**	305	609	914 (63.25%)
**Presence**	205	326	531 (36.75%)
**Total**	510	935	1455
**Relative risk (RR)** 1.09			
**CI** (1.00–1.186)			
***p* value** 0.045			

*CRC* colorectal cancer

On a multivariate analysis of demographic (age, gender) and clinical parameters (familiarity for diverticula and adenoma/CRC), only age was significantly associated with the development of colorectal adenomas or cancer (OR 1.05, 95% CI 1.03–1.07, *p* < 0.0001; Table 6).

**Table 6:** Multivariate analysis with multiple logistic regression and odds ratio (OR) adjusted for age, gender, family history for CRC, family history for diverticula, and presence of diverticula to study the independent association with adenomas and adenocarcinomas

**Variables**	**Odds ratio (CI 95%)**	***p* value**
**Age**	1.05 (1.03–1.07)	< 0.0001
**Sex** ** Female** ** Male**	1 (reference) 1.18 (0.74–1.88)	0.476
**Familiarity for CRC** ** Absent** ** Present**	1 (reference) 0.80 (0.49–1.32)	0.388
**Familiarity for diverticulosis** ** Absent** ** Present**	1 (reference) 0.67 (0.14–3.22)	0.620
**Diverticulosis** ** Absent** ** Present**	1 (reference) 1.05 (0.64–1.72)	0.834

*CRC* colorectal cancer

## Discussion

In recent years, several studies have attempted to evaluate the association between diverticulosis and colonic neoplastic lesions; however, these are quite heterogeneous due to different study designs, different types of patients included (concerning sex, age, clinical conditions, the severity of disease), and different comorbidities and indications to colonoscopy.

The data reported in our study showed a prevalence of 35.11% (527/1501) of asymptomatic diverticulosis and a prevalence of 47.04% (706/1501) of polyps among asymptomatic patients who underwent screening or surveillance colonoscopy. Interestingly, the multivariate analysis with logistic regression and odds ratios adjusted for age, gender, family history for CRC, family history for diverticula, and presence of diverticula revealed that the association between the presence of diverticula and development of neoplastic lesions (adenoma or carcinoma) depends on age: there is a threefold increased risk in over 60 patients compared to younger patients (OR 3,06, CI 95% 1.88–4.93), with a 5% increased risk for each year of age.

A recent meta-analysis involving 29 studies showed an increased risk of developing adenomas (OR 1.47, 95% IC 1.18–1.84) and polyps (OR 1.95, 95% IC 1.15–3.31), but not CRC (OR 0.98, 95% IC 0.63–1.50), in patients with colonic diverticula [29]. A sub-analysis showed that there is no increased risk of developing adenomas (OR 1.34, IC 95% 0.87–2.06) in patients with diverticulosis undergoing screening colonoscopy: this could be related to the young age of patients undergoing screening colonoscopy for CRC [29].

Accordingly, a cross-sectional study by Kieff et al. reported that the prevalence of colorectal polyps in patients with diverticulosis was significantly higher in patients over 60 years old and in female patients [30].

Therefore, even a high number of diverticula seems to be a risk factor for the development of CRC: in fact, this study showed an increased risk of CRC, located both distal (34.6% vs 16.3%; *p* = 0.03, 23.1% vs 5.7%; *p* = 0.003) and proximal (30.8% vs 14.9%; *p* = 0.049, 11.5% vs 4.3%; *p* = 0.13) in female subjects with extended distal diverticulosis compared to women with no or few distal diverticula. However, the overall comparison did not show statistically significant differences in this sense [30].

In this cross-sectional study, the association between colonic diverticula and the development of colorectal adenomas or cancer was statistically significant without finding associations with any type of polyp (including inflammatory and hyperplastic) or cancer. This could be related to the chronic inflammation involving the mucosa between the diverticula. This hypothesis is supported by the fact that most of the diverticula were found in the distal colon, the same site in which the neoplastic lesions were mainly found [30].

In agreement with this assumption, another study showed a threefold increase in cellular proliferation index in patients with asymptomatic diverticulosis compared to healthy controls [37].

According to the meta-analysis performed by Lee et al., a recent cross-sectional study by Tomaoglu including 3496 patients reported a significant relationship between diverticulosis and advanced adenoma polyps (> 10 mm, high-grade dysplasia, invasive cancer) (*p* < 0.05) but not with CRC (*p* = 0.232) [38].

Conversely, a statistically significant association between colonic diverticula and the development of polyps (including inflammatory, hyperplastic, and adenomatous) and cancer was reported in a retrospective study conducted by Viscido et al. (RR 2.67 CI 95% 2.27–3.15, *p* < 0.0001) [39].

A Sweden case–control study with a 14-year observation period (1992–2006) evaluated the risk of developing CRC in hospitalized patients with diverticular disease: the study was conducted on 41,037 patients and did not show an increased risk of development of CRC in diverticular disease [31].

Similarly, the study by Meurs-Szojda et al. did not show, through age-stratified analysis, a higher incidence of polyps (*p* = 0.478), CRC (*p* < 0.0001) and invasive adenocarcinoma (*p* = 0.0002) in patients with colonic diverticulosis; moreover, there is not an increased risk of polyps and CRC in patients with diverticulitis [33]. Finally, a prospective study reported no increased risk of developing adenomas (OR 1.0, 95% CI 0.7–1.4) or advanced adenomas (OR 0.8, 95% CI 0.4–1.5) in patients with colonic diverticulosis [32].

However, no studies with a prospective design showed a cause-effect relationship.

Moreover, a population-based study by Cooper et al. reported that in patients with CRC diagnosis, an associated diagnosis of diverticulosis was significantly most documented in patients with interval cancer (defined as patients with negative colonoscopy in 6–36 months before diagnosis) than in patients with a diagnosis of cancer (defined as patients with one colonoscopy within 6 months before diagnosis) (*p* = 0.001). Furthermore, diagnosis of diverticulosis was associated with interval cancers in all segments of the colon (proximal OR 2.88, 95% CI 2.66–3.12; distal OR 3.56, 95% CI 3.09–4.11; rectum OR 4.07, 95% CI 3.34–4.95) [40].

Our study has some limitations. The indications for surveillance colonoscopy due to a history of polyps, a family history of CRC, and colonic diverticula were largely self-reported by patients.

The robustness of the association of diverticulosis in cancer patients may be hindered by the limited number of CRCs detected in our study.

However, the main limitation is the study design; as a cross-sectional study, it was not possible to establish either the causal relationship between diverticula and colon cancer or the future risk of developing CRC in presence of diverticulosis. The relationship with the advanced age at the multivariate analysis suggests that the association between potential malignant (adenoma) or malignant lesions (carcinoma) and diverticulosis could be linked to a shared epidemiological factor (advanced age). However, as suggested by Cooper and colleagues’ alternative mechanisms including biological factors should be considered [40].

Diverticula and neoplastic lesions of the colon are frequently found during endoscopic examinations.

This study showed a statistically significant association between diverticula and colonic adenomas. However, it is impossible to establish a cause-effect relationship due to the study design. A study with a prospective design including both patients with diverticulosis and without colonic diverticula aimed at establishing the incidence of adenoma and CRC could be the best way to answer this relevant clinical question. In particular, a potential association could indicate the need for closer endoscopic surveillance.

## Data Availability

Data will be made available on reasonable request.
